# Melatonin as a regulator of apoptosis in leukaemia: molecular mechanism and therapeutic perspectives

**DOI:** 10.3389/fphar.2023.1224151

**Published:** 2023-08-14

**Authors:** Alireza Mafi, Hamidreza Rismanchi, Yasaman Gholinezhad, Mohaddese Malek Mohammadi, Vahide Mousavi, Seyed Ali Hosseini, Yaser Eshaghi Milasi, Russel J. Reiter, Behrooz Ghezelbash, Malihe Rezaee, Amirhossein Sheida, Fatemeh Zarepour, Zatollah Asemi, Mohammad Ali Mansournia, Hamed Mirzaei

**Affiliations:** ^1^ Department of Clinical Biochemistry, School of Pharmacy and Pharmaceutical Sciences, Isfahan University of Medical Sciences, Isfahan, Iran; ^2^ Nutrition and Food Security Research Center, Isfahan University of Medical Sciences, Isfahan, Iran; ^3^ School of Medicine, Shahid Beheshti University of Medical Sciences, Tehran, Iran; ^4^ Department of Pharmacology, School of Medicine, Shahid Beheshti University of Medical Sciences, Tehran, Iran; ^5^ School of Medicine, Bushehr University of Medical Sciences, Bushehr, Iran; ^6^ School of Medicine, Babol University of Medical Sciences, Babol, Mazandaran, Iran; ^7^ Department of Cell Systems and Anatomy, UT Health Long School of Medicine, San Antonio, TX, United States; ^8^ Department of Immunology, School of Medicine, Isfahan University of Medical Sciences, Isfahan, Iran; ^9^ Tehran Heart Center, Cardiovascular Diseases Research Institute, Tehran University of Medical Sciences, Tehran, Iran; ^10^ School of Medicine, Kashan University of Medical Sciences, Kashan, Iran; ^11^ Research Center for Biochemistry and Nutrition in Metabolic Diseases, Institute for Basic Sciences, Kashan University of Medical Sciences, Kashan, Iran; ^12^ Department of Epidemiology and Biostatistics, School of Public Health, Tehran University of Medical Sciences, Tehran, Iran

**Keywords:** melatonin, apoptosis, leukaemia, signalling pathway, therapy

## Abstract

Leukaemia is a dangerous malignancy that causes thousands of deaths every year throughout the world. The rate of morbidity and mortality is significant despite many advancements in therapy strategies for affected individuals. Most antitumour medications used now in clinical oncology use apoptotic signalling pathways to induce cancer cell death. Accumulated data have shown a direct correlation between inducing apoptosis in cancer cells with higher tumour regression and survival. Until now, the efficacy of melatonin as a powerful antitumour agent has been firmly established. A change in melatonin concentrations has been reported in multiple tumours such as endometrial, hematopoietic, and breast cancers. Findings show that melatonin’s anticancer properties, such as its prooxidation function and ability to promote apoptosis, indicate the possibility of utilizing this natural substance as a promising agent in innovative cancer therapy approaches. Melatonin stimulates cell apoptosis via the regulation of many apoptosis facilitators, including mitochondria, cytochrome c, Bcl-2, production of reactive oxygen species, and apoptosis receptors. This paper aimed to further assess the anticancer effects of melatonin through the apoptotic pathway, considering the role that cellular apoptosis plays in the pathogenesis of cancer. The effect of melatonin may mean that it is appropriate for use as an adjuvant, along with other therapeutic approaches such as radiotherapy and chemotherapy.

## 1 Introduction

Leukaemia is a type of malignancy that arises from the abnormal differentiation of hematopoietic cells and results in the large-scale proliferation of leukemic cells in the bone marrow, blood circulation, and lymph nodes (Juliusson and Hough, 2016). Despite improvement in the prognosis of patients with leukaemia because of novel progress in treatment strategies, it is still a life-threatening malignancy (Strati et al., 2018; Thol and Ganser, 2020). As reported by GLOBOCAN, leukaemia is the 15th highest new-case-detected cancer and the 11th highest cause of cancer mortality, with an estimated 474,519 cases and 311,594 deaths worldwide in 2020 (Sung et al., 2021). Two types of leukaemia, acute myeloid leukaemia (AML) and acute lymphoblastic leukaemia (ALL) are the most common cancers in children and bring about mortality and morbidity cancer-related causes in patients under 16 years old (Seth and Singh, 2015). Radiation, contact with specific chemicals, genetic mutations, familial positive history, and lifestyle are predisposing factors for leukaemia (Bispo et al., 2020). According to the World Health Organization (fifth edition) categorization of hematolymphoid tumours, the common classification of leukaemia is based on cell lineage, including lymphoid and myeloid, and the first category includes B-cell lymphoma and T/NK-cell lymphoma. Leukaemia can also be classified through the maturity of cells, clinical manifestations or genetic characterizations, and other subtype categories. Acute lymphoblastic leukaemia (ALL), acute myeloid leukaemia (AML), chronic lymphoid leukaemia (CLL), and chronic myeloid leukaemia (CML) are the four main kinds of leukaemia (Li and Li, 2022). Treatment for leukaemia has been improved over the years, and recent research focuses on targeted therapy and individualized treatment strategies (Sharma and Rai, 2019; Fleischmann et al., 2021). In addition to the type of leukaemia, leukocyte count, symptoms, and age as risk factors for treatment, epigenetic characterizations, immunologic profile, and assessment for treatment response are recently changed elements in the planning treatment programs for each patient (Lee and Cho, 2017; Kato and Manabe, 2018). Despite advances, lack of response to treatment, adverse effects of chemotherapy, illness recurrence, and medication resistance are the main concerns that stand in the way of leukaemia (Sharma and Rai, 2019; Zhang et al., 2019; Bárcenas-López et al., 2021).

N-acetyl-5-methoxy tryptamine, or melatonin, is an indoleamine that is secreted via the pineal gland, which was discovered for the first time in 1958 (Lerner et al., 1958; Hardeland et al., 2006). The rhythmic secretion of melatonin is controlled by hypothalamic suprachiasmatic nuclei, resulting in an increase in the level of melatonin in response to darkness (Vasey et al., 2021). Melatonin is distributed to blood and other body fluids and plays a protective role for the body by regulating homeostatic metabolisms with a concentration of 5–200 pg/mL per day (Hickie and Rogers, 2011). Melatonin is synthesized in different organs, including the retina, skin, gastrointestinal tract lymphocytes, and bone marrow (Acuña-Castroviejo et al., 2014). In addition to its circadian rhythm regulatory effect, melatonin has other functions, such as antioxidant, immunomodulatory, and oncostatin activity (Mafi et al., 2023a). The beneficial role of melatonin in the inhibition of the formation of various diseases has been proven for diabetes (Karamitri and Jockers, 2019), heart disease (Sun et al., 2016), neurodegeneration (Prodhan et al., 2021), depression (Tonon et al., 2021), and reproductive pathologies (Carlomagno et al., 2018). Melatonin has anticancer features through the induction of mechanisms including apoptosis, oxidation, and stop cell cycle and also inhibition of processes like angiogenesis, metastasis, and energy production (Reiter et al., 2018). Considering these mechanisms, the influence of melatonin in leukaemia, ovarian cancer, breast cancer, colorectal cancer, prostatic cancer, and others has been shown (Reiter et al., 2017; Kubatka et al., 2018; Mirza-Aghazadeh-Attari et al., 2020; Shafabakhsh et al., 2020; Shen et al., 2021). In addition, melatonin’s adjuvant properties, when used with other chemotherapy drugs, have been widely described (Mafi et al., 2023b). Studies have shown that melatonin has a preserving impact on lymphocytes and other hematopoietic cells, and promising responses in combination with chemotherapy and melatonin have been seen in leukemic patients (Rubio et al., 2007; Casado-Zapico et al., 2011; Lee et al., 2013). Thus, a comprehensive inspection of the antitumour pathways of melatonin is essential to utilize this hormone in leukaemia therapy. This essay aims to analyse and summarise the significance of melatonin in leukaemia’s apoptotic process.

## 2 Apoptosis: mechanisms in the cell

**FIGURE 1:**
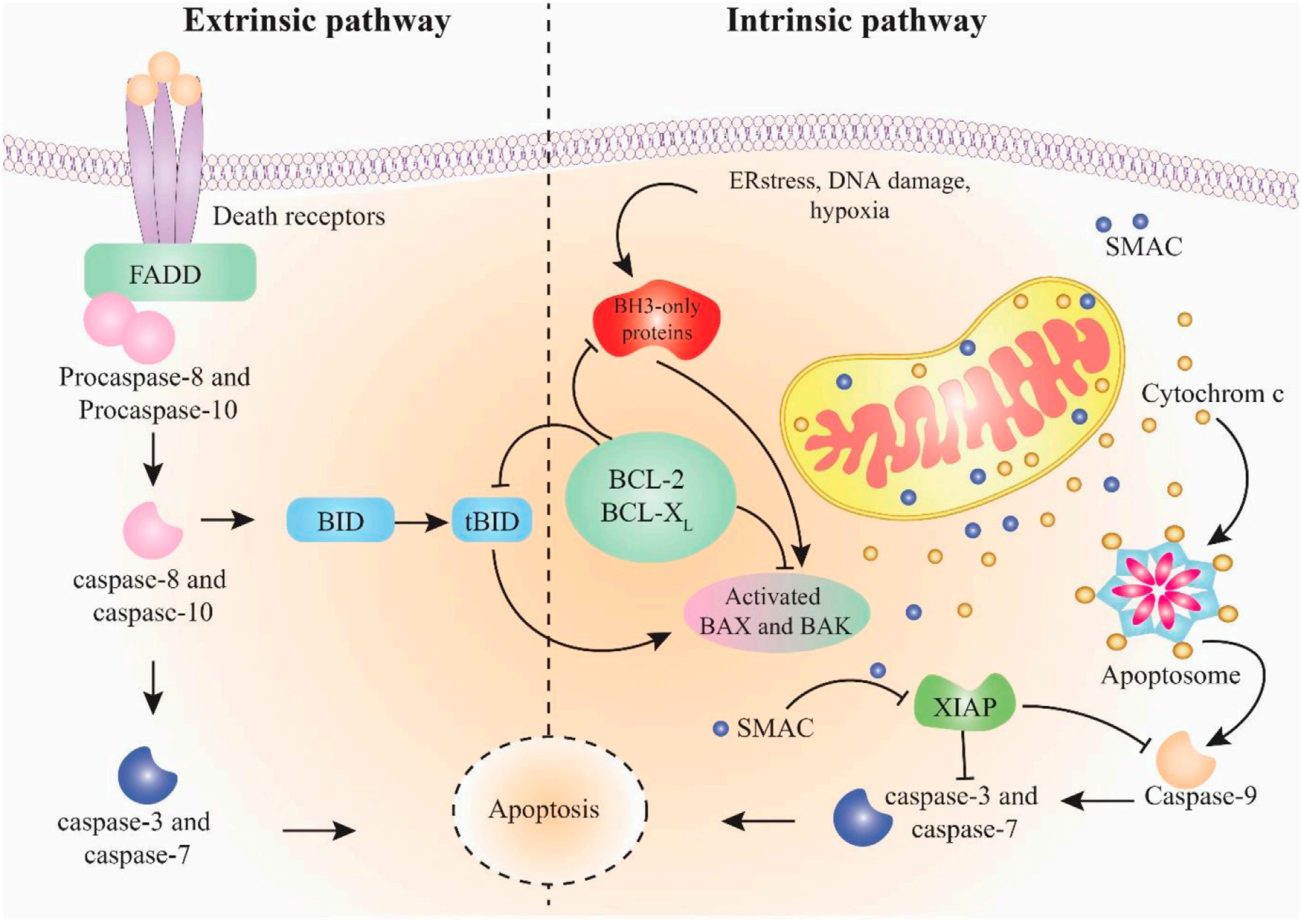
Schema of intrinsic and extrinsic pathways of apoptosis. The extrinsic pathway starts with the stimulation of death receptors followed by activation of caspase-8 and -10. The intrinsic pathway initiates in response to cell damage and continues by activation of caspase-9. Both pathways join in caspase-3 activation, which results in apoptosis.

Apoptosis is programmed cell death that differs from other types of cell death like necrosis. In apoptosis, the cell pursues a suicidal process that results in special morphological and biochemical alterations in the cell (Figure 1) (Wong, 2011; Bertheloot et al., 2021). Apoptosis is an essential biological mechanism in the regulation of embryonic development, differentiation and proliferation, homeostasis, and elimination of damaged and unwanted cells (Elmore, 2007; Voss and Strasser, 2020; Yaghoobi et al., 2023). Dysregulation of apoptosis is associated with uncontrolled cell proliferation and accumulation of mutated cells, eventually developing and progressing the cancer and also the resistance to cancer therapy (Fulda, 2009; Garg et al., 2018). Therefore, exploring the molecular components of apoptotic pathways is crucial when developing therapeutic targets and decreasing resistance during cancer treatment (Gerl and Vaux, 2005; Giménez-Bonafé et al., 2009).

The apoptotic mechanism occurs in two main pathways: intrinsic and extrinsic (Wong, 2011). Less known pathways include the apoptosis-like activation of the endoplasmic reticulum pathway (Szegezdi et al., 2006) and the perforin/granzyme pathway involved by granzyme A (GzmA) and granzyme B (GzmB) (Osińska et al., 2014). Eventually, these pathways enter into the common execution pathway of apoptosis (Kist and Vucic, 2021). The extrinsic apoptosis pathway begins with the external activation of death ligands binding to death receptors. Death receptors like tumour necrosis factor receptors (TNFR), Fas receptors, and TNF-related apoptosis-inducing ligand receptors (TRAILR) are found on the surface of cells (Contassot et al., 2007). Fas-associated death domain (FADD) and TNF receptor-associated death domain (TRADD) are two examples of intracellular domains used by death receptors (Boatright and Salvesen, 2003). Coherence of death ligands to death receptors (TNFα/TNFR1, (CD95, APO-1)/FasL, and TRAIL/TRAILR (DR5)) leads to revealing the death domain (DD) and binding to cell adaptors, which results in the fabrication of death-inducing signalling complex (DISC) (Seyrek et al., 2020). DISC begins the caspase-8 activation (member of the cysteine-protease family (Fritsch et al., 2019)). Finally, the activated caspase-8 enzyme starts the caspase cleavage and activates caspase-3 and inducts apoptosis through the execution pathway (Stennicke et al., 1998). The intrinsic pathway begins when the cell goes under stimulation, such as hypoxia, oxidative stress, genetic defects, or hyperaccumulation of intracellular Ca^2+^ (Kroemer et al., 2007). These provocations lead to disrupting the balance among proapoptotic proteins (like Bax, Bcl-Xs, and Bak) as well as antiapoptotic proteins (like Bcl-XL and Bcl-2) of the Bcl-2 family that are present in the mitochondrial membrane (García-Sáez, 2012). The predominance of proapoptotic protein impact alters the mitochondrial membrane outer membrane permeability (MOMP) (Dadsena et al., 2021). These modifications allow the mitochondrial inter-membrane gap to transfer cytochrome C into the cytoplasm and, in combination with Apaf-1 and caspase-9, develop a compound called apoptosome that continues the process through the caspase-3 activation and entrance in the execution pathway (Dorstyn et al., 2018). Additionally, the direct inhibitor of apoptosis proteins binding protein with low pI (DIABLO) and the second mitochondria-derived activator of caspase (Smac) are other components released from the perturbed mitochondrial membrane that link to the inhibitor of apoptosis proteins (IAP) and result in caspase’s separation to IAP. This mechanism causes the caspase-9 and -3 activations (Chai et al., 2000; Derakhshan et al., 2017). The common pathway is the last part of apoptosis that initiates via caspase-3 activation. Caspase-3 is responsible for the cleavage of proteins that are responsible for cytoskeletal construction and DNA repair. As a result, nuclear apoptosis and defects in signalling pathways occur, which eventually result in the cell becoming fragmented (Wong, 2011).

Alterations in every component of the mechanisms discussed previously can be associated with the reduction of apoptosis and resistance in cancer cells and the promotion of carcinogenesis. Pathological alterations defined include the death receptor signalling defect (Müschen et al., 2002), downregulation of caspase (Shen et al., 2010), variation in balance among proapoptotic and antiapoptotic proteins (Krajewska et al., 1996), and the loss of function of p53 because of gene mutations (Shen et al., 2010).

## 3 Melatonin

Melatonin plays a crucial role in different biological pathways in mammals. Because of its high lipid and water solubility, it can cross all layers of a cell and the cell’s nucleus and acts as a chemical mediator. Melatonin functions as a hormone, synchronizing the circadian rhythms of many organs and their functions (Alonso-Vale et al., 2008). Melatonin functionality can be classified into two major categories: receptor-mediated and non-receptor-mediated actions (Amaral et al., 2019). The latter can be defined as an interaction between melatonin and intracellular molecules, such as the antioxidative effects and scavenging reactive oxygen species (ROS). The double bonding in the carbon atoms of the melatonin structure gives it a high reduction ability, making it a powerful antioxidative agent. By directly scavenging free radicals or by subtly activating endogenous antioxidants, melatonin may reduce the effects of oxidative stress (Tiwari et al., 2021). Another example is the contact with enzymes like quinone reductase 2 and especially, type II calcium-calmodulin kinase, which melatonin directly inhibits. Through its impact on MT1 and MT2 receptors, melatonin synchronizes circadian rhythms, namely, the sleep–wake cycle and body temperature cycles (Liu et al., 2016). Furthermore, melatonin acts on MT3 receptors with several physiological activities, including the detoxification of free radicals along with antioxidant effects that protect the brain from oxidative stress (Hardeland, 2018). Melatonin exhibits both direct and indirect immunomodulatory effects, making it a potential pro- or anti-inflammatory agent in various conditions. Melatonin could stimulate the production of inflammatory cytokines and other inflammatory factors. Melatonin increases T-helper immune responses. Melatonin may act as an indirect immunoregulatory agent by decreasing nitric oxide formation, which eases the decrease of the inflammatory response. Many studies have shown the oncostatic property of melatonin in cancers such as colon, prostate, ovarian, oral, gastric, and breast cancers (Li et al., 2017a). The anticancer impacts of melatonin were observed to be mediated via various mechanisms, especially via antiapoptotic, antioxidative, and immunomodulatory signalling pathways, which all play an essential part in the growth and evolution of tumours (Reiter et al., 2018; Bhattacharya et al., 2019).

## 4 Melatonin induces apoptosis in cancer cells via different cellular pathways

Melatonin’s mechanism of action in inducing apoptosis has been examined in different types of cancer. These studies have consistently demonstrated that melatonin exerts its effects by suppressing cell proliferation and inducing apoptosis through the regulation of crucial signalling pathways involved in the apoptotic cascade (Majidinia et al., 2017).

It has been revealed that melatonin can exert a prooxidant effect on tumour cell lines, as evidenced by its ability to stimulate intracellular ROS production in human promyelocytic leukaemia cells. This prooxidant activity of melatonin has been associated with cytotoxic and proapoptotic outcomes. The significant increase in intracellular ROS production serves as a potential mechanism that could facilitate the apoptotic death of tumour cells (Bejarano et al., 2011). Melatonin’s antioxidant effects are the consequence of several intricate mechanisms, such as the activation of antioxidant enzymes and the prooxidant enzymes’ suppression, the inhibition of mitochondrial radical production, and the scavenging of free radicals (Hardeland et al., 2003).

Most of the positive outcomes associated with the administration of melatonin can be attributed to its impact on mitochondrial physiology (Acuna-Castroviejo et al., 2007; Acuña Castroviejo et al., 2011). Studies have shown that melatonin acts upon mitochondria and regulates the mitochondrial permeability transition pore (mPTP). The fabrication of mPTP results from high concentrations of Ca^2+^ or due to oxidative stress; it is also regarded as the first apoptosis stage (Halestrap, 2005). Ca^2+^ causes the pores to open, which depolarizes the inner membrane, interrupts the phosphorylation and respiration procedures, and causes the mitochondria to enlarge. This process also triggers the release of proapoptotic factors (Halestrap, 2009).

Multiple research studies have indicated a correlation between the suppression of p53 and the avoidance of apoptosis by both malignant and pre-malignant cells, resulting in the beginning and the advancement of tumorigenesis (Brown and Wouters, 1999; Yamamoto et al., 1999). The advancement of both the intrinsic and extrinsic routes of apoptosis depends heavily on p53. It is essential for controlling FasL and triggering caspase-8 that then causes Bid activation (Malki et al., 2006). By activating Bax as a result of the translocation of Bid to the mitochondrial membrane, cytochrome c is made more accessible for release (Schuler et al., 2000; Haupt et al., 2003). In contrast, activation of p53 can potentially lead to the inhibition of Bcl-2, an antiapoptotic protein regulated by p53 and located downstream of AKT (Miyashita et al., 1994). p53 promotes the activation of the caspases and the progression of extrinsic apoptosis by suppressing IAPs (Fridman and Lowe, 2003). Alonso-González et al. demonstrated that radiation exposure promotes the activation of p53, and treatment with melatonin enhances its effects (Alonso-González et al., 2016). Both endogenous DNA damage and chemotherapy-induced DNA damage are repaired more quickly when p53 is activated (Santoro et al., 2012; Santoro et al., 2013). Melatonin is a direct phosphorylating agent of p53, according to descriptions of its actions, resulting in the stimulation of DNA repair processes (Santoro et al., 2012). Melatonin action is inhibited by blocking melatonin receptors, and that inhibition can negatively impact the p53-dependent DNA repair reaction (Santoro et al., 2013). Melatonin treatment might increase p53 activity and promote late apoptosis (Alonso-González et al., 2018).

Research has indicated that the activation of MAPKs is highly correlated with the onset and growth of several forms of cancer. JNK, ERK, and p38 are only a few of the subfamilies that control MAPK signalling, which plays a role in regulating vital processes like apoptosis and proliferation via contact with pathways like PI3K and AKT (Burotto et al., 2014; Johnson et al., 2014). Additionally, p53’s proapoptosis activity may be impacted by MAPKs (Brown and Benchimol, 2006). Recently, many studies have focused on melatonin’s dual impact on the MAPK cascades and the PI3K/AKT axis in both cancerous and healthy cells. In normal cells, melatonin activates the AKT pathway, providing neuroprotective properties. However, when melatonin is administered to cancer cells, it inhibits the same pathway (Anhe et al., 2004; Klement et al., 2012; Kim et al., 2014). Several experimental studies have demonstrated that through the suppression of MAPK genes, melatonin can cause apoptosis and lessen the resilience of some cancer cells. However, in some cancers like SGC7901 gastric cancer cells, melatonin might upregulate MAPKs, and it might prevent cell proliferation through additional signalling mechanisms (Li et al., 2017b). Additionally, melatonin might boost redox activity, causing cell death, by inhibiting NFκB and activating MAPKs (Li et al., 2016).

PI3K is essential for many kinds of cancer cells to resist apoptosis. Survivin and other apoptosis inhibitors are activated by PI3K upregulation, making anticancer therapies less effective (Asechi et al., 2010). By focusing on the PI3K pathway, melatonin helps some cancer cells undergo apoptosis and lose viability. Melatonin can decrease the expression of IAP proteins like survivin, cIAP-1, XIAP, and cIAP-2 via decreasing PI3K (Fan et al., 2013). Melatonin was able to inhibit AKT phosphorylation in LoVo and SW480 cells when they were given 5-FU and melatonin, according to the research. Additional analyses showed that PI3K-specific inhibitor LY294002 selectively inhibits PI3K, which increases the suppression of cell viability, demonstrating the critical function of this pathway in limiting the growth of tumour cells. Downregulation of the PI3K/AKT pathway in the cancer cells described is correlated with an increase in PARP and proapoptotic caspases, according to *in vivo* xerography research (Gao et al., 2016).

## 5 Modulation of the apoptosis pathway by melatonin in leukaemia

Autophagy is an intracellular process in which damaged organelles or proteins are hydrolyzed by lysosomal enzymes. This process could both have prosurvival and promortality effects on cancer cells. On one hand, it can maintain cancer cell survival by hindering the caspase-dependent apoptosis, and it can lead to programmed cell death with over-activating of autophagy (Mizushima and Komatsu, 2011; Mariño et al., 2014; Chen et al., 2016). Several studies examined the potential role of melatonin on apoptosis through modulating autophagy in tumoral cells (Mehrzadi et al., 2021). For instance, in cervical cancer, adding melatonin to HeLa cells under cisplatin treatment enhances the function of the damaged mitochondrial membrane and hinders mitophagy through blockade of the JNK/Parkin pathway. Mitophagy is a process that preserves mitochondrial quality and homeostasis by selectively removing damaged mitochondria (Tan and Wong, 2017). The effect of melatonin on mitophagy leads to increased cytochrome-c releases from the mitochondrial membrane and activation of the caspase-9-dependent apoptosis pathway (Chen et al., 2018). The role of melatonin in inducing apoptosis through modulating autophagy in leukaemia has been examined. In one potential pathway, Lomovsky et al. showed that the combination therapy of melatonin and navitoclax in acute promyelocytic leukaemia increases the endoplasmic reticulum stress and release of autophagy-related proteins like protein kinase R (PKR)-like endoplasmic reticulum kinase (PERK). The increase in PERK levels increases the expression of C/EBP homologous protein (CHOP). The expression of CHOP is associated with activation of the apoptosis cascade by controlling antiapoptotic and proapoptotic proteins (Fernández et al., 2015). Moreover, CHOP promotes the release of Ca^2+^ from the endoplasmic reticulum. Changes in the Ca^2+^ capacities of cells affect the mitochondrial membrane permeability, which leads to ROS formation and initiation of apoptosis (Lomovsky et al., 2020a).

Acute promyelocytic leukaemia (APL) is a subtype of acute myeloid leukaemia (AML) that is different in the morphologic, clinical, and genetic aspects (Ghiaur et al., 2022). Wei et al. have illustrated a substantially lower APL cell viability when using arsenic trioxide (ATO), an approved drug for APL, together with melatonin, compared with either melatonin or ATO used separately (Wei et al., 2019).

ATO-induced apoptosis can occur through caspase signalling. Many proteins, including Bcl-2, an antiapoptotic, and Bax, a proapoptotic protein, are crucial in controlling caspase-dependent apoptosis (Wei et al., 2019). In the combination of ATO + melatonin, the caspase-9, caspase-3, and Bax expressions have been substantially increased. However, the level of prosurvival Bcl-2 proteins was lower in the ATO + melatonin group than in the ATO group. In addition, the ATO + melatonin group showed increased caspase-3 cleavage by regulating ATG-7-related autophagy. As a result, the apoptosis rate was higher while using melatonin as an adjuvant therapy combination with ATO than while using ATO alone. These findings all show that melatonin modifies autophagy to increase the cytotoxicity brought on by ATO in APL cells (Wei et al., 2019).

Mixed lineage leukaemia-rearranged (MLL-r) leukaemia occurs in 5%–10% of pediatric acute leukaemia cases with poor prognosis, relapsing, and therapeutic failure (Feng et al., 2016; Khaw et al., 2016). Human telomerase reverse transcriptase (hTERT) and COX-2 have been expressed in MLL-r leukaemia, and their expression levels have roles in tumorigenicity and poor prognosis (Calvello et al., 2018). The COX-2 signalling pathway might enhance the growth, metastasis, and invasion of cancer cells (Woo et al., 2015).

Melatonin has the potential to regulate the COX-2 and hTERT signalling pathways. RBFOX3 (RNA-binding protein fox-1 homolog 3) is one of the transcription factors located on the hTERT promoter region that regulates hTERT expression. It has been seen that melatonin significantly reduced RBFOX3’s ability to bind to the hTERT promoter and repressed the production of the hTERT protein. In addition, melatonin hindered the RBFOX3 expression in MLL-r cell lines, and, in lower RBFOX3 expression, a significant reduction in the hTERT expression was seen (Tang et al., 2019).

In many malignancies, the binding of numerous transcription factors, including NF-κB p65, to the COX-2 promoter region controls COX-2 production (Lu et al., 2016; Shrestha et al., 2017). It was observed that melatonin reduced the NF-κB p65 expression in the nucleus of the MLL-r cell lines. Moreover, melatonin prevented NF-κB p65 from attaching to the COX-2 promoter. As a result, melatonin could inhibit the NF-κB p65/COX-2 signalling pathway in the MLL-r cell lines. Furthermore, melatonin has increased caspase-9 and caspase-3 activities in the MLL-r cell lines. Therefore, melatonin could induce leukemic cell apoptosis by enhancing the caspase-dependent apoptotic pathway. Tang et al. compared the melatonin-treated mice group with the control group and stated that melatonin reduces the expression of the proteins such as RBFOX3, COX-2, and hTERT and lowers the growth of cancer cells *in vivo* (Tang et al., 2019).

Acute myeloid leukaemia (AML) is characterized by the most prevalent genetic alteration, the type III receptor Fms-like tyrosine kinase 3 (FLT3) mutation, which affects around one-third of AML patients (Kennedy and Smith, 2020). Despite it being a renowned antioxidant, some cancer cells are also made to produce ROS by melatonin (Florido et al., 2022). Overproduction of ROS causes mitochondrial malfunction, which eventually results in the death of cancer cells (Chen et al., 2020). It has been discovered that melatonin caused FLT3/ITD human leukaemia cells to undergo apoptosis via transferring cytochrome c from the mitochondria into the cytosol. As was mentioned in earlier studies, mitochondrial cytochrome c is a key molecule that causes apoptosis in cells (Tian et al., 2019; Xiang et al., 2019).

Sorafenib is a treatment strategy that has the potential to kill leukaemia blasts with FLT3/ITD (Bae et al., 2022). Sorafenib exerts its effect by blocking tyrosine receptor signalling and inducing ROS in tumour cells (Wan et al., 2013). When melatonin and sorafenib are used together, more cytochrome c is transferred from mitochondria into the cytosol and more intracellular ROS are accumulated than when either drug is used alone. Melatonin thus increases sorafenib’s anticancer effects in FLT3/ITD cells. According to Tian et al., melatonin and sorafenib together showed much stronger proapoptosis and antiproliferation actions than either drug alone, *in vitro* and *in vivo*. Melatonin and sorafenib together led to greater leukaemia cell depletion in peripheral blood smears and greater reductions in spleen and liver weight in mice as compared to mice treated with sorafenib alone (Tian et al., 2019).

5′-Adenosine monophosphate-activated kinase (AMPK) is a cellular enzyme that regulates energy homeostasis (Dengler, 2020). It was stated in numerous studies that activation of this enzyme could regulate apoptosis. Thereby, the therapeutic target AMPK for cancer may be worth considering (Jiang et al., 2020; Dehnavi et al., 2021). The combination of melatonin with puromycin, an unselective anticancer drug, increased AMPK’s phosphorylation in HL-60 human APL cells. As a result, melatonin enhanced puromycin-induced apoptosis by raising the AMPK activation in HL-60 cells. In addition, this resulted in an increase in caspase-3 activation and the cleavage of poly ADP-ribose polymerase (PARP), both of which are crucial for HL-60 cells to undergo apoptosis (Koh et al., 2011). DNA strand nicks and breaks stimulate PARP activation. In minimal DNA damage, PARP is a survival factor that causes DNA repair. In a high-DNA damage condition, PARP induces apoptosis (Cherian et al., 2008). In addition to the caspase-3 activation, melatonin synergistically hindered the expression of the antiapoptotic proteins Bcl-x_L_ and Bcl-2 (Koh et al., 2011).

Melatonin was observed to cause apoptosis in HL-60 cells by depolarizing the mitochondrial membrane, inducing permeability transition pores, and activating caspase-9 and -3. Cytochrome c was transferred into the cytosol as a result of permeability transition pore and mitochondrial membrane depolarization stimulation. Additionally, melatonin may greatly increase the expression of proapoptotic proteins like Bax and Bid in the leukaemia HL-60 cell lines (Bejarano et al., 2009).

Cytarabine is an antitumor drug that is utilized in APL therapy, non-Hodgkin lymphoma (NHL), and chronic myelocytic lymphoma (CML) (Liao et al., 2021). Lomovsky et al. demonstrated that melatonin substantially increased the effects of cytarabine when they were used together. Using cytarabine and melatonin together could reduce the mitotic index (MI) and the level of Bcl-2 more than using cytarabine alone (Lomovsky et al., 2020b).

An important factor for the beginning of apoptosis is the formation of the mitochondrial permeability transition pore (mPTP) in the inner mitochondrial membrane. After the mPTP is formed, the outer mitochondrial membrane becomes more permeable, causing the mitochondria to enlarge and release cytochrome c into the cytosol from the inter-membrane gap (Lee and Lee, 2018; Aslam et al., 2021). The voltage-dependent anion channel (VDAC) and the translocator protein (TSPO) are factors that play a role in mPTP regulation (Bayrhuber et al., 2008; Lee et al., 2020). According to reports, the quantity of VDAC is said to increase in certain tumours, making it a potential target for anticancer treatments (Mathupala and Pedersen, 2010). In addition, it has been seen that the level of TSPO increased in many tumours and played a substantial role in the development of cancer cells. Therefore, TSPO expression could be considered a tumorigenicity agent (Veenman et al., 2004; Wu and Gallo, 2013; Shehadeh et al., 2019).

It is reported that low (10^–6^–10^–8^ M) concentrations of melatonin increased intracellular glutathione, which is an antioxidant agent in the cells, and ultimately enhanced cell viability. In contrast, in high (10^–3^–10^–4^ M) concentrations, melatonin would induce the production of intracellular ROS and the reduction of glutathione, which contribute to apoptosis induction in the leukaemia CMK, HL-60, Jurkat, and MOLT-4 cell lines (Büyükavcı et al., 2006; Büyükavci et al., 2011; Estaras et al., 2021).

Radiotherapy irradiations influence both tumour cells and normal cells via ROS production induction and DNA damage that leads to apoptosis (Qin et al., 2021). When exposed to radiation, the tumour suppressor gene p53 is activated, which causes apoptosis (Sisakht et al., 2020). Activated p53 induces cell cycle arrest to give damaged DNA a repair opportunity or promotes apoptosis to prevent the propagation of cells with damaged DNA (Feroz and Sheikh, 2020). Jang et al. compared the radiation-induced apoptosis in mouse normal and leukaemia cells treated with melatonin with control groups. They observed that in the irradiated normal cells that were incubated with melatonin, the expression of p53 was reduced; in contrast, Bcl-2 expression increased compared with control groups. As a result, melatonin had the potential ability to reduce radiation-induced apoptosis in normal cells. In contrast, they found different results in the leukaemia cells. Melatonin enhanced the expression of the p53 protein in the melatonin-treated, irradiated-leukaemia cells compared to controls. Therefore, melatonin increased radiation-induced apoptosis in leukaemia cells, unlike normal cells (Jang et al., 2009).

**FIGURE 2:**
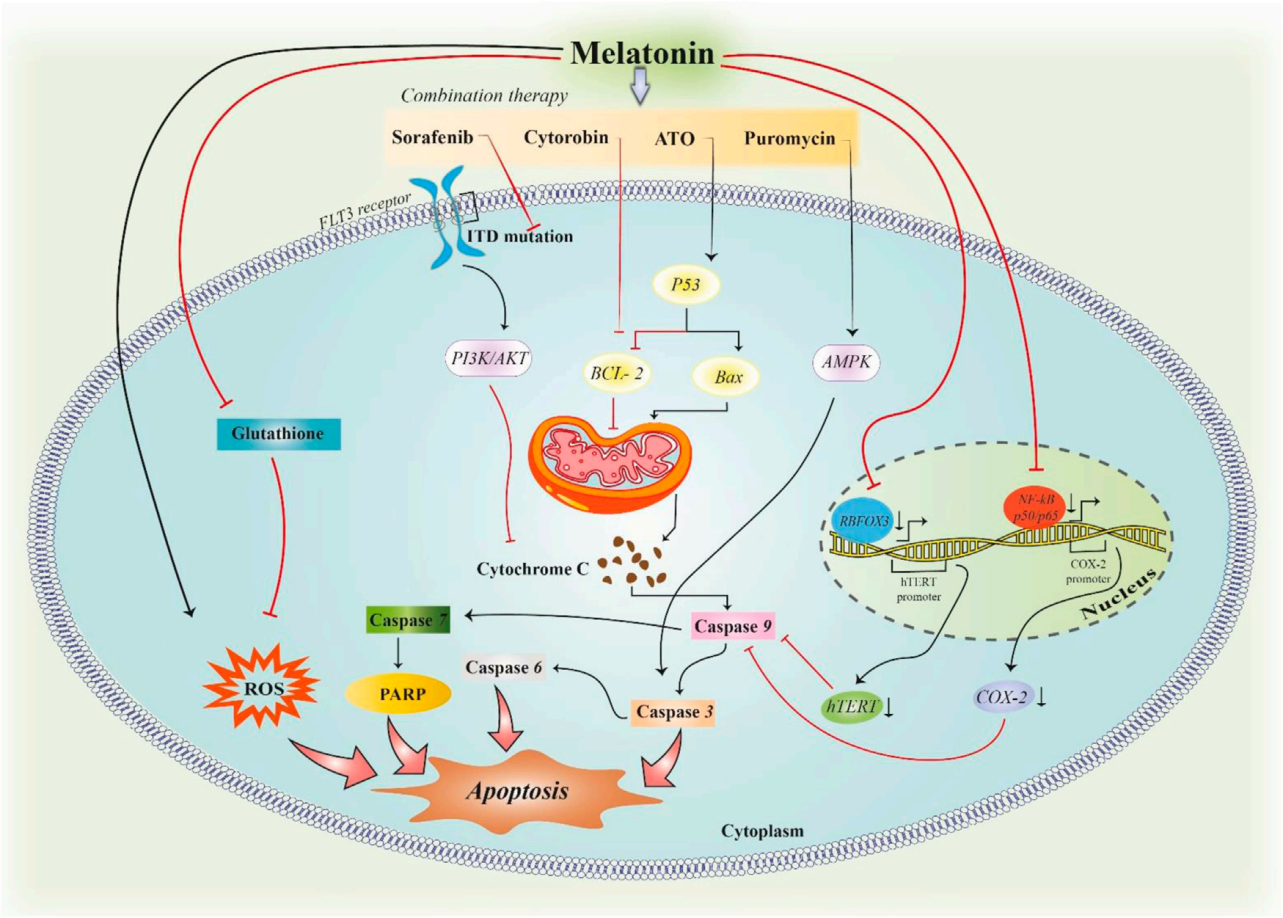
Induction of apoptosis in leukaemia by melatonin. Melatonin can induce apoptosis in leukaemic cells by inducing ROS formation. In addition, melatonin can downregulate the expression of Cox-2 and hTERT genes, which leads to hindering the inhibitory effect of these two proteins on the caspase-dependent pathway of apoptosis. The combination therapy of melatonin with antitumour drugs in leukaemia can synergize the induction of apoptosis in tumoral cells. Each pathway modulates the function of proteins in the waterfall of apoptosis.

Perdomo et al. evaluated the probable potential of melatonin in apoptosis of Molt-3 leukaemia cells, and interestingly, they found that melatonin significantly enhances the activation of caspase-3, -6, -7, and -9 but only found slight enhancement in caspase-8. Therefore, they suggested that melatonin induced apoptosis by the intrinsic pathway in Molt-3 cells. As the Bcl-2 protein family is an important agent to control the intrinsic pathway via mitochondria outer membrane permeability control, they evaluated this family and realized that melatonin induced the upregulation of proapoptotic factor Bax while it reduced Bcl-2 and Bcl-XL, two prosurvival proteins that are expressed contrarily. As a result, melatonin increased the Bax/Bcl-2 ratio that was contributing to apoptosis induction (Perdomo et al., 2013). Collectively, the summarized information shows that melatonin alone and/or together with chemotherapeutic agents can induce apoptosis in leukaemia through different signalling pathways (Figure 2).

## 6 Synergistic cytotoxicity of melatonin and anticancer drugs in leukaemia

Studies demonstrated that melatonin has a dose-dependent cytotoxicity potential. Zhelev et al. evaluated the leukaemia cytotoxic feature of several anticancer drugs in solo applications compared to melatonin additive usage. It has been found that melatonin has a greater cytotoxic capacity to kill leukaemia cells when combined with anticancer medications, including everolimus, lonafarnib, palbociclib, MG-132, bortezomib, and barasertib, than when these medications were used separately (Lee et al., 2013).

APL is principally defined by the chromosomal translocation of the gene of the retinoic acid receptor-alpha (RARA) (Mannan et al., 2020). Retinoic acid is mainly used in the treatment of APL, but it has adverse effects on the human body (Afacan Ozturk et al., 2021; Zhang et al., 2022). The simultaneous use of melatonin with retinoic acid enhances its effects on HL-60. Therefore, a lower dose of retinoic acid is needed in the combination therapy than the current common therapeutic dose; subsequently, its side effects are reduced (Krestinina et al., 2018). According to a study conducted by Krestinina et al., the combined impact of melatonin and retinoic acid with a much lower content than that used for retinoic acid alone previously has similar effects on the reduction of the antiapoptotic Bcl-2 protein and VDAC expressions. It also caused a further decline in TSPO expression and, thus, tumorigenicity suppression (Krestinina et al., 2018).

Everolimus is an immunosuppressant drug that also possesses anticancer activity through inhibition of the mammalian target of the rapamycin (mTOR) pathway that is important for controlling cell viability, translation start, and cell cycle progression (Ramírez-Valle et al., 2010; Zhu et al., 2020). Everolimus had a slight cytotoxic effect on leukaemia lymphocytes through the induction of apoptosis. It has been observed that melatonin enhanced the cytotoxic effect of everolimus by exhibiting a strong induction of apoptosis, although not on normal lymphocytes. This apoptosis was not dependent on ROS production because the level of ROS was reduced when melatonin was added to everolimus compared to the use of everolimus alone (Zhelev et al., 2017). It might occur because of the downregulation of mTOR expression and other oncogenes due to melatonin (Lee et al., 2013).

Based on the Buyukavci et al. study, melatonin did not enhance the cytotoxic effects of antileukaemia medications like cytarabine, etoposide, and daunorubicin in leukaemia cells *in vitro*. However, melatonin has protective potential on normal cells against chemotherapeutic drugs *in vitro* (Büyükavci et al.). For instance, melatonin could reduce the cardiac cytotoxicity side effect of daunorubicin by its indirect antioxidant activity (Zare et al., 2021).

When combined with chemotherapy and radiotherapy, hyperthermia is a powerful approach to treating cancer. Hyperthermia has the potential to decrease cell growth and cause alterations in the nuclei of the cells that are exposed to it, which leads to apoptosis. (Kang et al., 2020; Zastko et al., 2021). Melatonin could enhance the number of apoptotic cells when this hormone is added to hyperthermia treatment, which is used on both U937 and HL-60 AML cell lines. Interestingly, the combination of melatonin with hyperthermia increased the caspase-9, -8, -3, and caspase-2 activation in the caspase-dependent pathway apoptosis and release of cytochrome c to the cytosol more than the use of hyperthermia alone (Quintana et al., 2016).

## 7 Conclusion and perspective

Various studies have proven that melatonin has a wide range of positive effects in the treatment of leukaemia by influencing several cellular and molecular pathways in leukaemia cells. Melatonin also improves the effects of other chemotherapy medications in combination. Melatonin’s proapoptotic and prooxidant characteristics, together with its powerful influence on DNA damage, are its key therapeutic benefits in the treatment of leukaemia. Melatonin can decrease DNA damage, improve DNA repair, and promote antioxidant enzymes in normal cells and tissues, particularly chemo/radiosensitive cells like bone marrow and lymphocytes. This may lessen apoptosis and improve tissue tolerance. Antiapoptotic genes like NF-B and Bcl-2 are often highly expressed in cancer cells, and, in contrast, p53 function may be reduced. Through the upregulation and activation of proapoptotic molecules, including p53 and Bax, melatonin plays a part in the control of cancer cell death. Melatonin plays a critical part in boosting ROS formation through mitochondria in different cancer cells. When used with different chemotherapeutic drugs, melatonin was indicated to increase superoxide and depolarize the mitochondrial membrane. An increase in the rate of mitophagy after melatonin administration has been proposed as an indicator for mitochondrial impairment and cell death. Melatonin has been found to enhance apoptosis via suppressing mitophagy in some cancerous cells. Overall, one of the most powerful properties of melatonin that can increase the sensitivity of tumour cells to treatment methods like chemotherapy and radiotherapy appears to be the regulation of apoptotic signalling pathways.
